# Clinicopathological predictors and an internally validated exploratory nomogram for 2-year recurrence after hepatic resection of hepatocellular carcinoma ≤5 cm

**DOI:** 10.3389/fmed.2026.1948479

**Published:** 2026-10-08

**Authors:** Wei Zhang, Yubo Zhang, Qi Wang, Peng Tian, Ruilong Bai, Peng Lei

**Affiliations:** Department of Hepatobiliary Surgery, General Hospital of Ningxia Medical University, Yinchuan, Ningxia, China

**Keywords:** hepatocellular Carcinoma, Ki-67, Microvascular invasion, preliminary nomogram, Recurrence-free survival, satellite nodules, small HCC

## Abstract

**Background:**

Recurrence remains common after resection of hepatocellular carcinoma (HCC) even when tumor diameter is limited. We evaluated routinely available clinicopathological predictors of recurrence within 2 years after resection of HCC ≤5 cm and developed an exploratory nomogram with bootstrap internal validation.

**Methods:**

This retrospective cohort included patients who underwent hepatic resection from 2014 to 2022. Two-year recurrence was analyzed using logistic regression, and recurrence-free survival (RFS) was analyzed using Cox regression. The revised full model contained seven explicitly specified predictors: Ki-67, preoperative TACE/RFA, microvascular invasion (MVI), satellite nodules, tumor diameter, prothrombin time (PT), and preoperative ascites. Missing model covariates were addressed in a 50-dataset multiple-imputation sensitivity analysis. Internal validation used 1,000 bootstrap resamples with the complete seven-predictor model refitted in each resample.

**Results:**

Among 215 patients, 93 (43.3%) developed recurrence within 24 months. The complete-case model included 190 patients and 83 events. In adjusted logistic analysis, Ki-67, preoperative TACE/RFA, MVI, satellite nodules, tumor diameter, and PT were associated with recurrence; preoperative ascites was retained but was not statistically significant. In adjusted Cox analysis, Ki-67, MVI, satellite nodules, and PT were associated with shorter 2-year RFS. The nomogram had an apparent AUC of 0.775 and an optimism-corrected AUC of 0.744; the optimism-corrected Brier score and calibration slope were 0.206 and 0.808, respectively. Multiple-imputation estimates were directionally consistent with the complete-case results.

**Conclusions:**

In patients with resected HCC ≤5 cm, proliferative activity, microscopic dissemination, tumor burden, and hepatic synthetic function contributed to early-recurrence risk. The internally validated nomogram showed moderate discrimination but remains exploratory and requires independent external validation before clinical use.

## Introduction

Hepatocellular carcinoma (HCC) remains one of the major causes of cancer-related mortality worldwide, and its management requires integration of tumor burden, liver function, and patient fitness (1–6). With the increased use of surveillance and cross-sectional imaging, a growing proportion of patients are diagnosed with tumors potentially amenable to curative-intent therapy.

Tumor size terminology is not uniform. A solitary HCC ≤2 cm corresponds to the very-early BCLC subgroup, whereas thresholds of ≤3 cm and ≤5 cm have also been used in surgical and transplant-oriented literature (3, 7–10). Recurrence nevertheless remains substantial: in a two-center Western cohort of HCC ≤2 cm, 29% recurred within 2 years (10), and an earlier series of HCC ≤3 cm reported 20 recurrences among 50 patients (40%) within 2 years (11). These data show that limited tumor size does not necessarily imply indolent biology.

Several pathological features, including MVI, satellite nodules, tumor size, and proliferative activity, have been associated with recurrence after hepatectomy (12–16). A recent nomogram developed in 326 patients with HCC ≤3 cm incorporated AFP, MVI grade, Edmondson–Steiner grade, resection type, and margin width and reported 2-year RFS AUCs of 0.856 and 0.874 in its training and split-sample validation sets, respectively (9). Rather than claiming a first nomogram, the present study examines whether continuous Ki-67 together with microscopic spread and liver-status measures provides a complementary, readily available postoperative risk profile in a broader ≤5-cm cohort.

We therefore defined the study population explicitly as pathologically confirmed HCC with a maximum tumor diameter ≤5 cm. This study-specific threshold was selected as a pragmatic upper boundary used in classic surgical/transplant criteria (7, 8), not as a substitute for the ≤2-cm very-early HCC convention. We aimed to evaluate factors associated with recurrence within 24 months, identify predictors of 2-year RFS, and construct an exploratory clinicopathological nomogram with internal validation.

## Methods

### Study design and patients

This was a retrospective cohort study of patients who underwent hepatic resection between 2014 and 2022 at the General Hospital of Ningxia Medical University. Reporting was planned with reference to observational-study and prediction-model reporting principles (17, 18). Patients were eligible if the final pathological diagnosis was HCC and the maximum tumor diameter was ≤5 cm. Patients with non-HCC malignancy, benign lesions, mixed or uncertain pathology, or HCC >5 cm were excluded from the final analysis.

### Definitions and endpoint

For clarity, the cohort is described throughout by its explicit maximum-diameter criterion rather than by a universal size label. Preoperative TACE or RFA was recorded as previous locoregional therapy; it was not interpreted as a randomized treatment exposure. MVI, satellite nodules, differentiation, capsule, vascular invasion, and immunohistochemical findings were extracted from pathology reports. Original differentiation descriptions were retrospectively mapped to well, moderate, or poor differentiation: explicitly well-differentiated tumors were coded as well; moderate and mixed well–moderate descriptions as moderate; and poor or mixed moderate–poor descriptions as poor. Histologic labels without an explicit grade and missing descriptions were retained as ungraded/unclear. Recurrence was diagnosed using imaging findings and/or comprehensive clinical assessment during follow-up.

The primary endpoint was 2-year RFS. RFS was calculated from hepatic resection to first documented recurrence. Recurrence within 24 months was coded as an event. Patients without recurrence within 24 months, including those who recurred after 24 months, were administratively censored at 24 months.

### Variables

Demographic and clinical variables included age, sex, cirrhosis, history of hepatitis, antiviral treatment, comorbidities, smoking, alcohol use, preoperative ascites, and preoperative TACE/RFA. Laboratory variables included complete blood count, liver biochemical parameters, alpha-fetoprotein (AFP), prothrombin time (PT), D-dimer, neutrophil-to-lymphocyte ratio (NLR), platelet-to-lymphocyte ratio (PLR), and systemic immune-inflammation index (SII). Perioperative and pathological variables included blood transfusion, postoperative ascites, intraoperative blood loss, operation time, tumor size, tumor number, Ki-67, differentiation, capsule status, MVI, satellite nodules, vascular invasion, nerve invasion, CD34, GPC-3, and AFP immunohistochemistry.

### Statistical analysis

Continuous variables are presented as median (IQR), and categorical variables as number and percentage. Between-group comparisons used the Mann–Whitney U test for continuous variables and Fisher's exact test or the chi-square test for categorical variables. For the recoded differentiation table, association with recurrence was tested among gradable cases using a Monte Carlo exact test with 100,000 simulated tables; the minimum expected cell count after collapsing was 12.35. Univariable logistic and Cox models evaluated Ki-67, preoperative TACE/RFA, MVI, satellite nodules, tumor diameter, albumin, PT, D-dimer, NLR, log10 AFP, preoperative ascites, and antiviral treatment. To eliminate ambiguity in the multivariable specification, the revised full model contained seven explicitly fixed predictors spanning proliferation, prior treatment, microscopic spread, tumor burden, hepatic synthetic function, and portal-hypertension status: Ki-67, preoperative TACE/RFA, MVI, satellite nodules, tumor diameter, PT, and preoperative ascites. All seven were retained regardless of adjusted *P* value; no automated stepwise selection or post-fit significance-based removal was used. Differentiation was not eligible for prediction modeling because the source reports did not use a standardized grading system and required retrospective harmonization.

A logistic-regression nomogram was constructed from the fixed seven-predictor model. Cox proportional hazards models used the Breslow method for tied event times. The primary analysis used complete cases for model covariates. Discrimination was quantified by the AUC, overall prediction error by the Brier score, calibration by grouped observed versus predicted risks and calibration slope/intercept, and potential clinical utility by decision-curve analysis (17, 19, 20). For internal validation, 1,000 bootstrap samples were drawn from the complete-case development set; the complete seven-predictor model was refitted in every resample, tested in the original sample, and mean optimism was subtracted from apparent performance.

Missingness was tabulated for every variable analyzed in Tables 1, 2. Patients with observed versus missing Ki-67 were compared using Mann–Whitney U or Fisher's exact tests, and a logistic trend model evaluated calendar-year ascertainment. As a sensitivity analysis, missing model covariates were multiply imputed in 50 datasets by fully conditional specification with predictive mean matching for Ki-67; the imputation model included recurrence status, Nelson–Aalen cumulative hazard, calendar year, and clinical covariates. Binary MVI and satellite-nodule values were imputed conditionally. Logistic and Cox estimates were combined using Rubin's rules. D-dimer was not imputed for multivariable modeling because it was not a fixed model predictor. Analyses were two-sided with *P* < 0.05 and were performed in Python 3.12.

**Table 1:** Baseline clinical characteristics and preoperative laboratory variables according to 2-year recurrence status in hepatocellular carcinoma ≤5 cm.

Variable	Overall	No recurrence at 24 months	Recurrence within 24 months	Statistic	*P* value
Demographic and clinical characteristics					
Male sex, n (%)	165 (76.7%)	94 (77.0%)	71 (76.3%)	Fisher	1.000
Age, years	55.00 (48.00-63.00)	55.00 (48.25-64.75)	54.00 (48.00-59.00)	MWU=6182.5	0.260
Cirrhosis, n (%)	179 (83.3%)	97 (79.5%)	82 (88.2%)	Fisher	0.100
History of hepatitis, n (%)	191 (88.8%)	106 (86.9%)	85 (91.4%)	Fisher	0.383
Antiviral treatment, n (%)	91 (42.3%)	46 (37.7%)	45 (48.4%)	Fisher	0.127
Hypertension, n (%)	60 (27.9%)	32 (26.2%)	28 (30.1%)	Fisher	0.543
Diabetes mellitus, n (%)	31 (14.4%)	18 (14.8%)	13 (14.0%)	Fisher	1.000
Smoking, n (%)	78 (36.3%)	42 (34.4%)	36 (38.7%)	Fisher	0.568
Alcohol use, n (%)	40 (18.6%)	20 (16.4%)	20 (21.5%)	Fisher	0.379
Preoperative TACE/RFA, n (%)	17 (7.9%)	4 (3.3%)	13 (14.0%)	Fisher	0.005
Preoperative ascites, n (%)	47 (21.9%)	21 (17.2%)	26 (28.0%)	Fisher	0.068
Preoperative laboratory variables					
WBC, ×10^9/L	4.79 (3.80-5.83)	4.55 (3.73-5.52)	5.10 (4.16-5.99)	MWU=4976.5	0.124
Neutrophils, ×10^9/L	2.50 (1.82-3.34)	2.28 (1.78-3.17)	2.68 (2.10-3.53)	MWU=4764.5	0.045
Lymphocytes, × 10^9/L	1.57 (1.28-1.96)	1.62 (1.30-1.98)	1.54 (1.28-1.93)	MWU=5920.5	0.585
Platelets, ×10^9/L	144.00 (100.50-184.00)	149.00 (99.75-191.25)	136.00 (101.00-176.00)	MWU=6120.5	0.323
AFP, ng/mL	27.85 (3.99-710.60)	20.83 (3.08-375.00)	34.87 (5.48-827.30)	MWU=5028.0	0.182
Albumin, g/L	38.50 (36.10-41.10)	38.90 (36.55-41.27)	38.10 (35.50-40.70)	MWU=6426.0	0.096
AST, U/L	28.50 (22.70-36.15)	27.30 (22.70-33.70)	30.40 (22.80-39.50)	MWU=4882.5	0.080
ALT, U/L	31.40 (23.60-45.45)	32.60 (23.00-46.65)	31.00 (23.60-44.20)	MWU=5615.5	0.900
Total bilirubin, μmol/L	15.90 (11.35-20.35)	16.06 (11.25-20.40)	15.60 (11.40-20.20)	MWU=5787.0	0.802
PT, s	12.20 (11.60-13.10)	12.05 (11.43-12.78)	12.50 (11.80-13.70)	MWU=4422.5	0.006
D-dimer	0.22 (0.13-0.42)	0.20 (0.12-0.33)	0.27 (0.17-0.45)	MWU=3295.5	0.016
NLR	1.53 (1.21-2.08)	1.42 (1.14-1.87)	1.64 (1.32-2.35)	MWU=4551.0	0.013
PLR	88.99 (71.11-115.30)	90.86 (70.54-117.02)	87.11 (72.09-107.81)	MWU=5803.0	0.774
SII	219.70 (145.06-332.70)	200.46 (139.60-309.61)	228.57 (153.56-335.75)	MWU=5180.0	0.276

Continuous variables are presented as median (IQR), and categorical variables as n (%). *P* values were calculated using Fisher's exact test for binary variables and the Mann–Whitney U test for continuous variables. Abbreviations: AFP, alpha-fetoprotein; ALT, alanine aminotransferase; AST, aspartate aminotransferase; IQR, interquartile range; MWU, Mann–Whitney U test; NLR, neutrophil-to-lymphocyte ratio; PLR, platelet-to-lymphocyte ratio; PT, prothrombin time; RFA, radiofrequency ablation; SII, systemic immune-inflammation index; TACE, transarterial chemoembolization; WBC, white blood cell count.

**Table 2:** Perioperative and pathological characteristics according to 2-year recurrence status in hepatocellular carcinoma ≤5 cm.

Variable	Overall	No recurrence at 24 months	Recurrence within 24 months	Statistic	*P* value
Perioperative variables					
Transfusion, n (%)	20 (9.3%)	11 (9.0%)	9 (9.7%)	Fisher	1.000
Postoperative ascites, n (%)	43 (20.0%)	20 (16.4%)	23 (24.7%)	Fisher	0.168
Intraoperative blood loss, mL	200.00 (100.00–400.00)	200.00 (100.00–400.00)	200.00 (150.00–400.00)	MWU=5279.0	0.379
Operation time, h	2.00 (2.00–3.00)	2.00 (2.00–3.00)	2.00 (2.00–3.00)	MWU=5271.0	0.416
First porta hepatis occlusion time, min	21.50 (15.00–30.00)	24.00 (16.50–29.00)	20.00 (14.00–31.00)	MWU=403.5	0.415
Pathological characteristics					
Tumor maximum diameter, cm	3.00 (2.00–4.00)	3.00 (2.00–4.00)	3.50 (2.50–4.00)	MWU=4732.5	0.036
Tumor number	1.00 (1.00–1.00)	1.00 (1.00–1.00)	1.00 (1.00–1.00)	MWU=5673.0	1.000
Ki-67, %	25.00 (10.00–40.00)	20.00 (10.00–30.00)	30.00 (15.00–50.00)	MWU=3194.0	<0.001
Differentiation, n (%)				Monte Carlo exact	0.010
Well	29 (13.5%)	21 (17.2%)	8 (8.6%)		
Moderate	106 (49.3%)	66 (54.1%)	40 (43.0%)		
Poor	67 (31.2%)	29 (23.8%)	38 (40.9%)		
Ungraded/unclear	13 (6.0%)	6 (4.9%)	7 (7.5%)		
Capsule present, n (%)	61 (28.5%)	33 (27.3%)	28 (30.1%)	Fisher	0.651
MVI positive, n (%)	62 (29.0%)	24 (19.8%)	38 (40.9%)	Fisher	0.001
Satellite nodules, n (%)	32 (15.0%)	9 (7.4%)	23 (24.7%)	Fisher	<0.001
Vascular invasion, n (%)	5 (2.3%)	1 (0.8%)	4 (4.3%)	Fisher	0.169
Nerve invasion, n (%)	1 (0.5%)	0 (0.0%)	1 (1.1%)	Fisher	0.429
CD34 positive, n (%)	175 (91.6%)	102 (94.4%)	73 (88.0%)	Fisher	0.121
GPC-3 positive, n (%)	143 (77.3%)	78 (73.6%)	65 (82.3%)	Fisher	0.214
AFP IHC positive, n (%)	66 (35.7%)	40 (37.7%)	26 (32.9%)	Fisher	0.537

Continuous variables are presented as median (IQR), and categorical variables as n (%). *P* values used Fisher's exact test or Mann–Whitney U test as appropriate. Differentiation was tested among the 202 gradable cases using a Monte Carlo exact test with 100,000 simulated tables; ungraded/unclear cases were excluded from that *P* value. Mixed well–moderate descriptions were mapped to moderate, and poor or mixed moderate–poor descriptions to poor. AFP IHC, alpha-fetoprotein immunohistochemistry; HCC, hepatocellular carcinoma; MVI, microvascular invasion.

## Results

### Patient selection and outcome distribution

A total of 223 records were screened. Six patients were excluded because of non-HCC, benign, or other pathology, and two were excluded because pathological maximum tumor diameter was >5 cm. The final cohort included 215 patients with HCC ≤5 cm. Recurrence within 24 months occurred in 93 patients (43.3%); 122 had no recurrence or were censored at 24 months (Figure 1).

**Figure 1:**
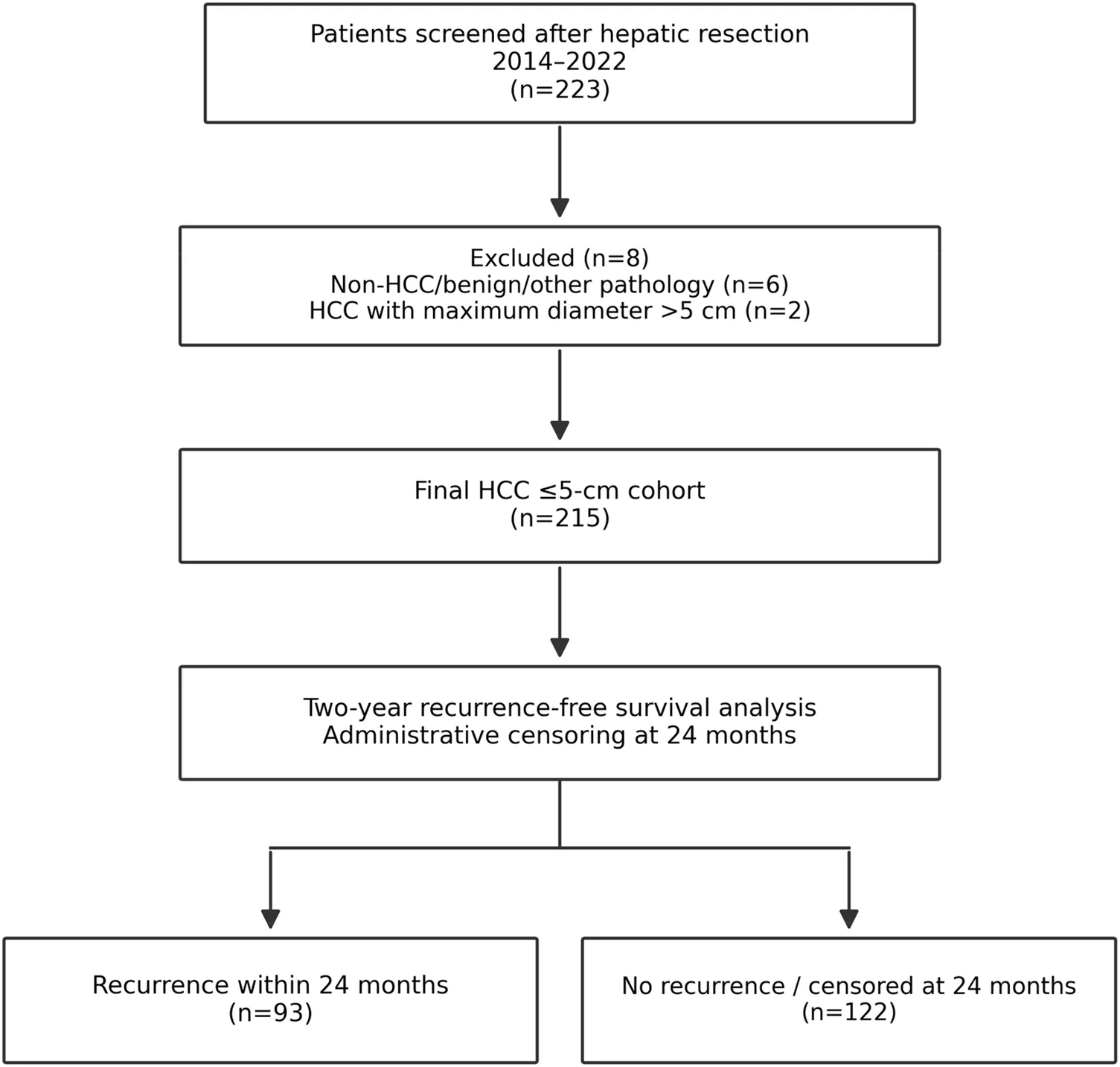
Study flowchart for the final cohort of hepatocellular carcinoma ≤5 cm. HCC, hepatocellular carcinoma.

### Baseline clinical and laboratory characteristics

Baseline characteristics according to 2-year recurrence status are shown in Table 1. The groups were similar with respect to age, sex, cirrhosis, hepatitis history, antiviral treatment, comorbidities, smoking, and alcohol use. Preoperative TACE/RFA was more frequent among patients with recurrence (14.0% vs 3.3%, *P* = 0.005). Patients with recurrence also had higher neutrophil counts, longer PT, higher D-dimer levels, and higher NLR.

### Perioperative and pathological characteristics

Perioperative and pathological findings are summarized in Table 2. Perioperative variables did not differ significantly between groups. Patients with recurrence had larger tumors, higher Ki-67, and higher frequencies of MVI and satellite nodules. After harmonization, differentiation was well in 29, moderate in 106, poor in 67, and ungraded/unclear in 13 patients. Among 202 gradable cases, differentiation was associated with recurrence (Monte Carlo exact *P* = 0.010). Because grading had not been recorded with a standardized system and 13 cases remained ungraded/unclear, differentiation was retained as a descriptive sensitivity variable and was not entered into the prediction model.

The distribution of key variables is shown in Figure 2. Patients with recurrence within 24 months had visibly higher Ki-67 and tumor diameter, as well as modestly higher inflammatory and coagulation markers reflected by NLR and log-transformed D-dimer.

**Figure 2:**
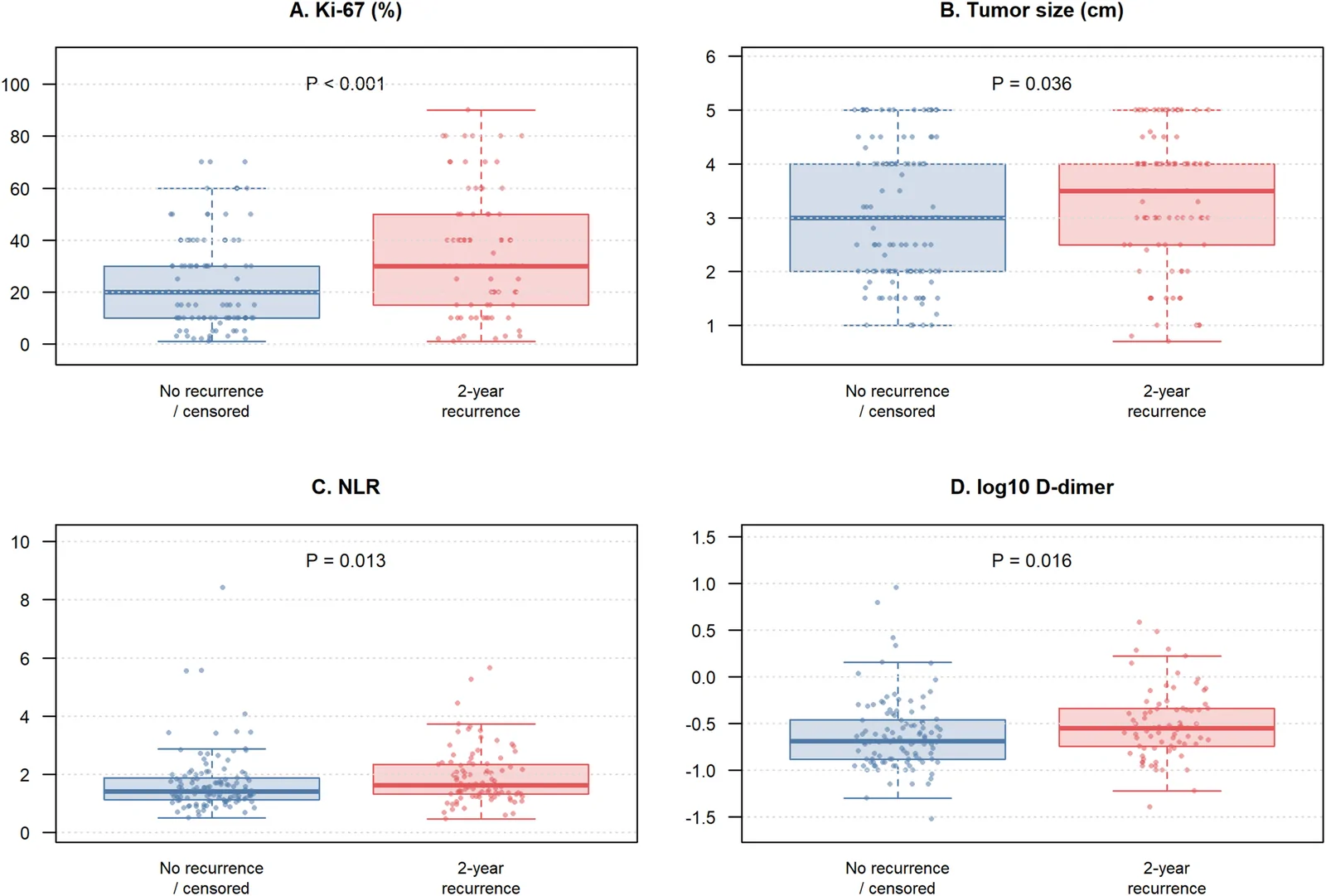
Comparison of key variables according to 2-year recurrence status in hepatocellular carcinoma ≤5 cm.

### Factors associated with recurrence within 24 months

In the fixed seven-predictor logistic model (Table 3), recurrence within 24 months was associated with Ki-67 (adjusted OR 1.028 per 1% increase, *P* = 0.001), preoperative TACE/RFA (OR 6.673, *P* = 0.012), MVI (OR 2.301, *P* = 0.027), satellite nodules (OR 2.871, *P* = 0.031), tumor diameter (OR 1.374 per cm, *P* = 0.031), and PT (OR 1.387 per second, *P* = 0.030). Preoperative ascites was retained in the full model but was not statistically significant (OR 1.566, *P* = 0.264).

**Table 3:** Univariable and fixed multivariable logistic regression for recurrence within 24 months after hepatic resection of hepatocellular carcinoma ≤5 cm.

Variable	N	Events	Univariable OR (95% CI)	Univariable statistic	Univariable *P* value	Adjusted OR (95% CI)	Adjusted statistic	Adjusted *P* value
Ki-67, per 1%	190	83	1.027 (1.012-1.042)	z = 3.53	<0.001	1.028 (1.011–1.044)	z = 3.30	<0.001
Preoperative TACE/RFA	215	93	4.794 (1.509-15.232)	z = 2.66	0.008	6.673 (1.529–29.117)	z = 2.53	0.012
MVI positive	214	93	2.792 (1.519-5.133)	z = 3.31	<0.001	2.301 (1.098–4.824)	z = 2.21	0.027
Satellite nodules	214	93	4.089 (1.789-9.344)	z = 3.34	<0.001	2.871 (1.099–7.497)	z = 2.15	0.031
Tumor size, per cm	215	93	1.261 (1.007-1.579)	z = 2.02	0.044	1.374 (1.030–1.834)	z = 2.16	0.031
Albumin, per g/L	215	93	0.945 (0.881-1.014)	z = -1.58	0.114			
PT, per s	215	93	1.434 (1.132-1.816)	z = 2.99	0.003	1.387 (1.033–1.861)	z = 2.18	0.030
D-dimer, per 1 unit	185	77	1.032 (0.752-1.415)	z = 0.19	0.846			
NLR, per 1 unit	215	93	1.253 (0.946-1.660)	z = 1.58	0.115			
AFP, per log10 ng/mL	214	93	1.157 (0.886-1.511)	z = 1.07	0.283			
Preoperative ascites	215	93	1.866 (0.972-3.585)	z = 1.87	0.061	1.566 (0.713–3.439)	z = 1.12	0.264
Antiviral treatment	215	93	1.549 (0.896-2.677)	z = 1.57	0.117			

The adjusted columns report the fixed full model containing Ki-67, preoperative TACE/RFA, MVI, satellite nodules, tumor diameter, PT, and preoperative ascites; all seven predictors were retained irrespective of adjusted *P* value. ORs >1 indicate higher odds of recurrence within 24 months. Abbreviations: CI, confidence interval; MVI, microvascular invasion; OR, odds ratio; RFA, radiofrequency ablation; TACE, transarterial chemoembolization.

### Predictors of 2-year recurrence-free survival

Kaplan–Meier curves showed poorer 2-year RFS in patients with Ki-67 ≥ 30%, MVI, satellite nodules, and preoperative TACE/RFA (Figure 3). In the fixed seven-predictor Cox model (Table 4), shorter 2-year RFS was associated with Ki-67 (adjusted HR 1.022 per 1% increase, *P* < 0.001), MVI (HR 1.763, *P* = 0.016), satellite nodules (HR 1.924, *P* = 0.016), and PT (HR 1.235 per second, *P* = 0.023). Preoperative TACE/RFA showed an attenuated risk estimate (HR 1.829, *P* = 0.071); tumor diameter (HR 1.171, *P* = 0.114) and preoperative ascites (HR 1.420, *P* = 0.188) were not statistically significant (Figure 4).

**Figure 3:**
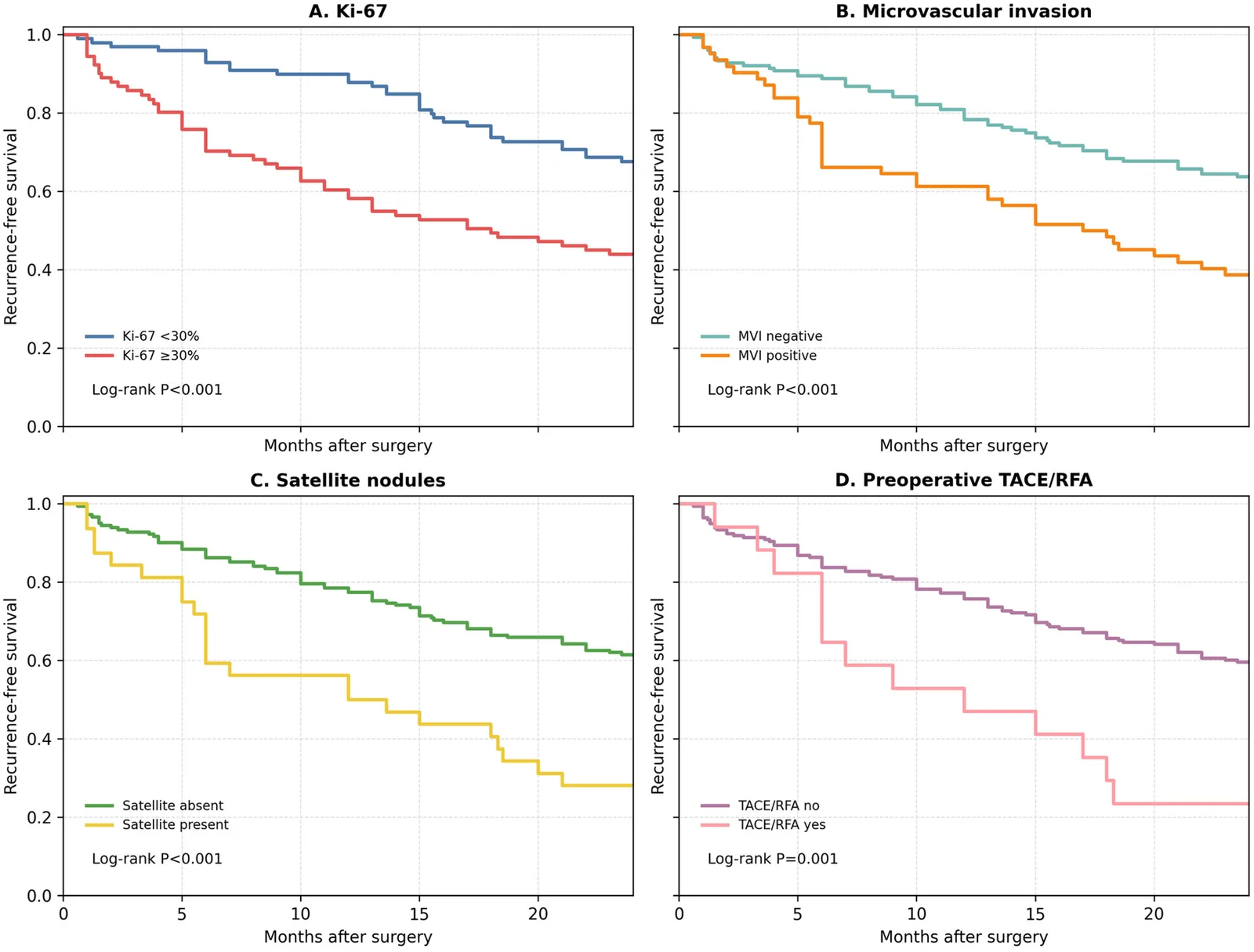
Kaplan–meier curves for 2-year recurrence-free survival, stratified by ki-67, MVI, satellite nodules, and preoperative tACE/RFA. Log-rank *P* values are shown.

**Figure 4:**
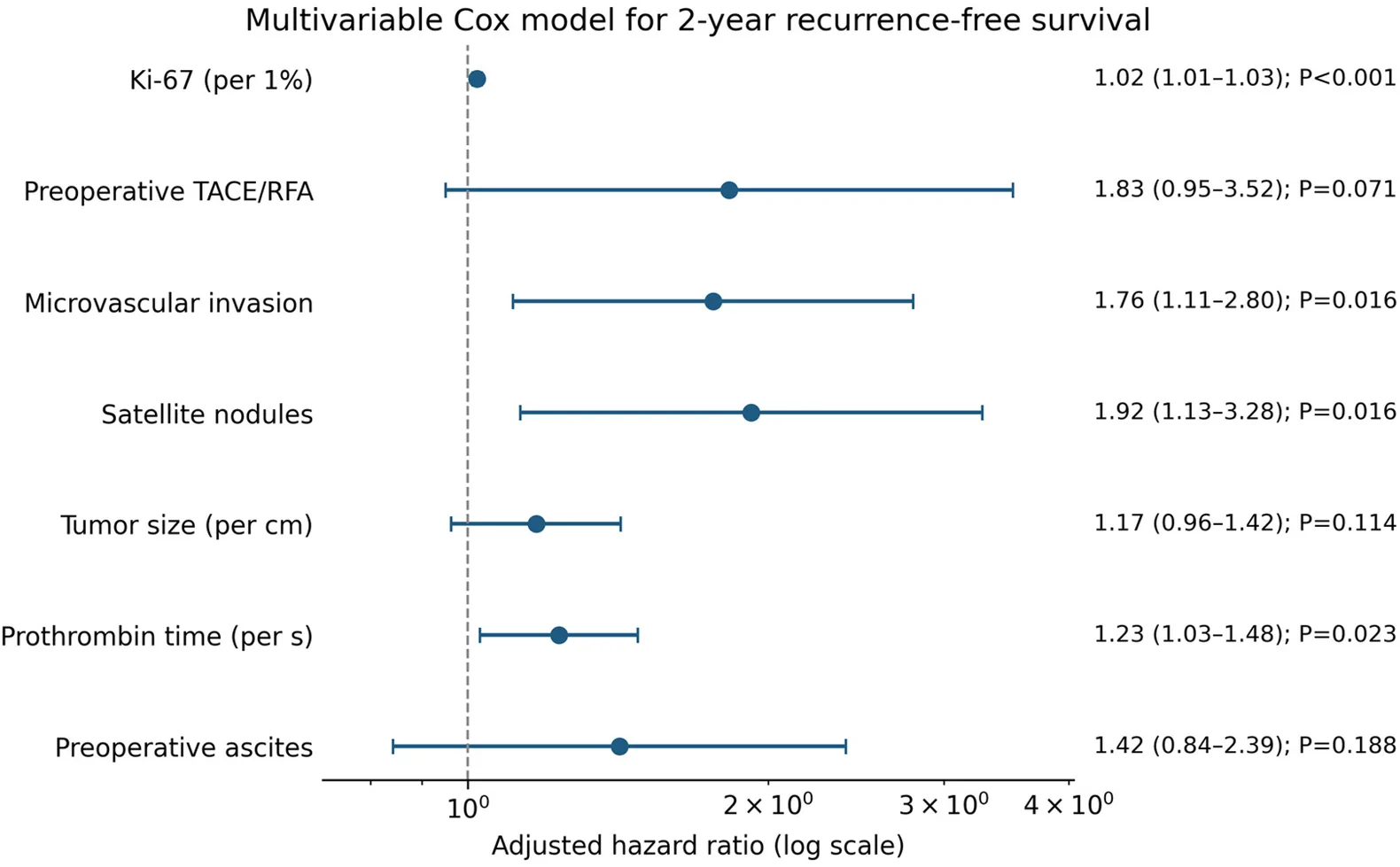
Fixed seven-predictor cox model for 2-year recurrence-free survival. Points show adjusted HRs and bars show 95% CIs.

**Table 4:** Univariable and fixed multivariable cox regression for 2-year recurrence-free survival in hepatocellular carcinoma ≤5 cm.

Variable	N	Events	Univariable HR (95% CI)	Univariable statistic	Univariable *P* value	Adjusted HR (95% CI)	Adjusted statistic	Adjusted *P* value
Ki-67, per 1%	190	83	1.023 (1.013-1.033)	z = 4.45	<0.001	1.022 (1.012–1.033)	z = 4.11	<0.001
Preoperative TACE/RFA	215	93	2.558 (1.419-4.612)	z = 3.12	0.002	1.829 (0.950–3.519)	z = 1.81	0.071
MVI positive	214	93	2.092 (1.382-3.166)	z = 3.49	<0.001	1.763 (1.111–2.796)	z = 2.41	0.016
Satellite nodules	214	93	2.568 (1.600-4.123)	z = 3.91	<0.001	1.924 (1.129–3.279)	z = 2.41	0.016
Tumor size, per cm	215	93	1.160 (0.980-1.374)	z = 1.72	0.085	1.171 (0.963–1.425)	z = 1.58	0.114
Albumin, per g/L	215	93	0.957 (0.910-1.006)	z = -1.74	0.082			
PT, per s	215	93	1.285 (1.092-1.512)	z = 3.02	0.002	1.235 (1.030–1.481)	z = 2.28	0.023
D-dimer, per 1 unit	185	77	1.038 (0.846-1.275)	z = 0.36	0.719			
NLR, per 1 unit	215	93	1.138 (0.968-1.338)	z = 1.56	0.119			
AFP, per log10 ng/mL	214	93	1.120 (0.917-1.367)	z = 1.11	0.267			
Preoperative ascites	215	93	1.588 (1.009-2.499)	z = 2.00	0.045	1.420 (0.842–2.394)	z = 1.32	0.188
Antiviral treatment	215	93	1.392 (0.927-2.091)	z = 1.59	0.111			

RFS was administratively censored at 24 months. The adjusted columns report the fixed seven-predictor model containing Ki-67, preoperative TACE/RFA, MVI, satellite nodules, tumor diameter, PT, and preoperative ascites. HRs >1 indicate shorter 2-year RFS/higher recurrence hazard. Abbreviations: CI, confidence interval; HCC, hepatocellular carcinoma; HR, hazard ratio; MVI, microvascular invasion; RFS, recurrence-free survival; RFA, radiofrequency ablation; TACE, transarterial chemoembolization.

### Nomogram and model performance

The revised nomogram incorporated all seven fixed predictors (Figure 5) and was fitted in 190 complete cases with 83 events. Apparent AUC was 0.775. Bootstrap-estimated AUC optimism was 0.031, yielding an optimism-corrected AUC of 0.744. The apparent and optimism-corrected Brier scores were 0.188 and 0.206, respectively; the optimism-corrected calibration slope was 0.808 and intercept 0.005. At the apparent Youden cutoff of 0.552, sensitivity was 0.578, specificity 0.888, and accuracy 0.753 (Table 5; Figure 6).

**Figure 5:**
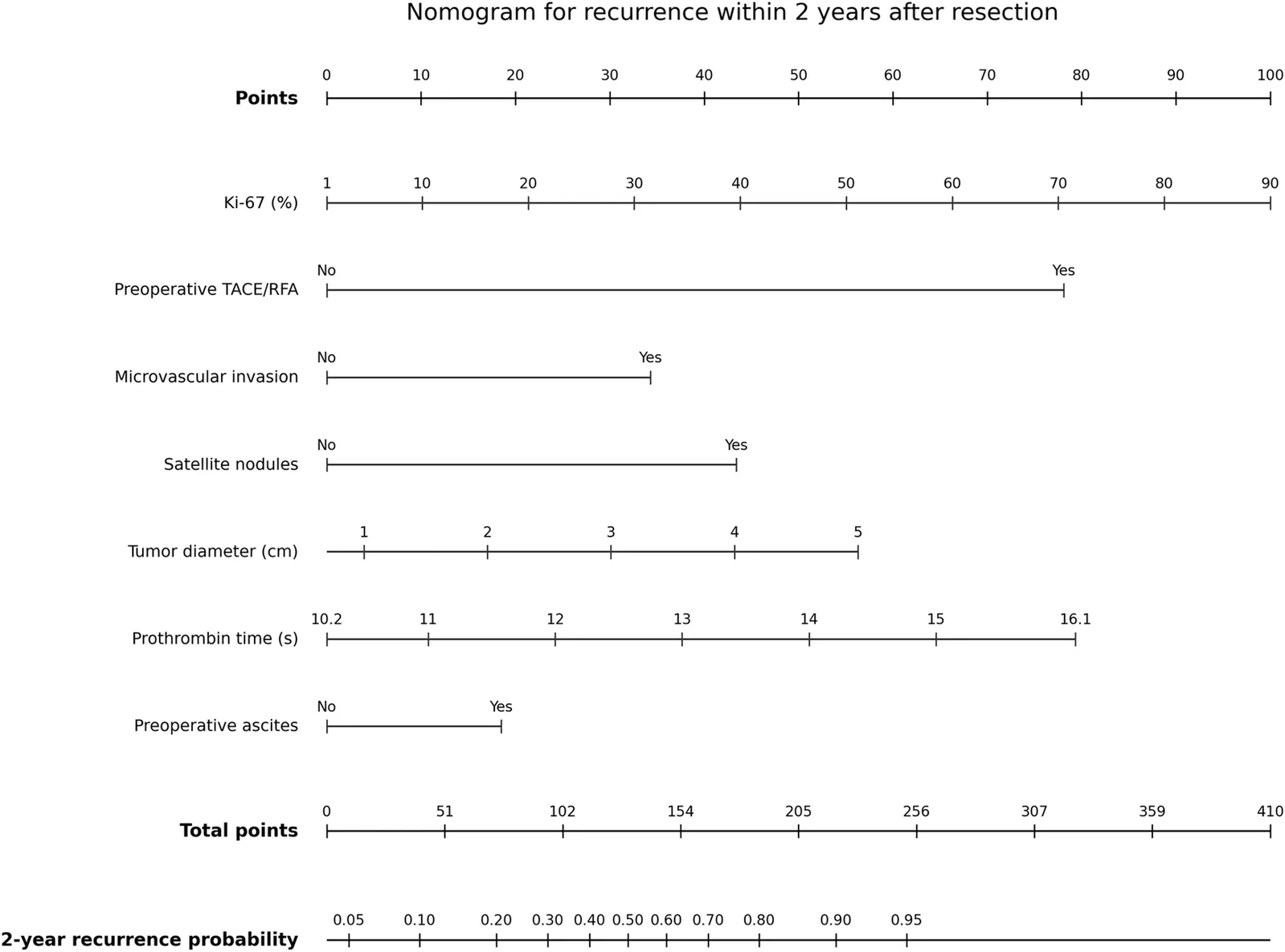
Revised nomogram for recurrence within 2 years after resection of hepatocellular carcinoma ≤5 cm. The fixed logistic model includes Ki-67, preoperative TACE/RFA, MVI, satellite nodules, tumor diameter, PT, and preoperative ascites. The nomogram remains exploratory pending external validation.

**Table 5:** Apparent and bootstrap optimism-corrected performance of the seven-predictor 2-year recurrence model.

Metric	Value
Patients included in model, n	190
2-year recurrence events, n (%)	83 (43.7%)
Fixed predictors, n	7
Apparent AUC	0.775
Bootstrap AUC optimism	0.031
Optimism-corrected AUC	0.744
Apparent Brier score	0.188
Optimism-corrected Brier score	0.206
Optimism-corrected calibration slope	0.808
Optimism-corrected calibration intercept	0.005
Apparent Youden cutoff	0.552
Sensitivity at apparent cutoff	0.578
Specificity at apparent cutoff	0.888
Accuracy at apparent cutoff	0.753
Successful bootstrap resamples	1000/1,000

The complete seven-predictor logistic model was refitted in each of 1,000 bootstrap resamples. Optimism equals bootstrap-sample performance minus performance when the resample-fitted model was applied to the original development sample; corrected performance equals apparent performance minus mean optimism. The Youden cutoff and classification measures are apparent descriptive estimates and were not used to define clinical risk groups.

**Figure 6:**
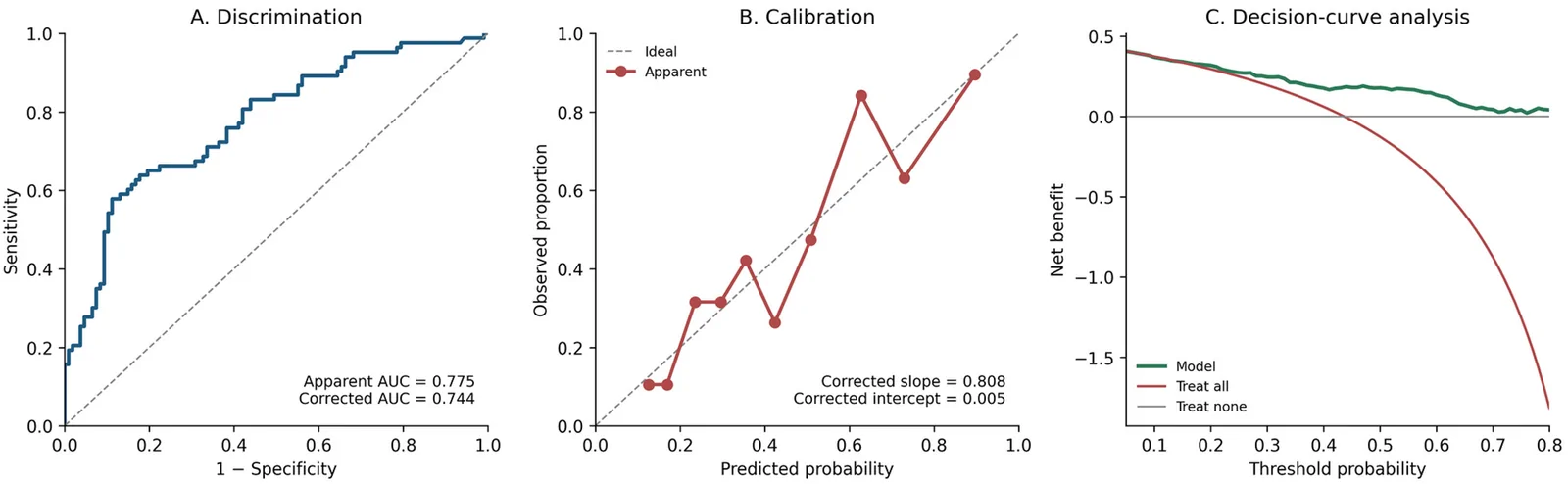
Performance of the fixed seven-predictor logistic model. **(A)** Apparent ROC curve with apparent and bootstrap-corrected AUCs. **(B)** Apparent grouped calibration with bootstrap-corrected calibration slope and intercept. **(C)** Apparent decision-curve analysis. Bootstrap correction used 1,000 resamples.

### Sensitivity analysis

After excluding the 17 patients who received preoperative TACE/RFA, the complete-case Cox sensitivity analysis included 175 patients. Ki-67, MVI, satellite nodules, tumor diameter, and PT remained associated with shorter RFS, whereas preoperative ascites did not (Supplementary Table S1). Missingness is summarized in Supplementary Table S2. Ki-67 was missing in 25/215 patients (11.6%); 24 had no other missing model covariate and one also lacked MVI and satellite-nodule status. Ki-67-missing patients were treated earlier (median year 2017 vs 2019, *P* = 0.012) and were younger (median 52 vs 55 years, *P* = 0.042), but their recurrence proportion, tumor diameter, MVI, satellite nodules, PT, and preoperative-treatment distribution did not differ materially (Supplementary Table S3). Missing Ki-67 decreased over calendar time (OR per year 0.798, 95% CI 0.670–0.951; *P* = 0.012), indicating a plausible temporal ascertainment mechanism. In 50 multiply imputed datasets, effect directions were consistent with the complete-case models, although several borderline *P* values shifted (Supplementary Tables S4 and S5).

## Discussion

In this cohort of resected HCC ≤5 cm, recurrence within 24 months occurred in 43.3% of patients. The revised analyses identified Ki-67, MVI, satellite nodules, and PT as consistent correlates of shorter 2-year RFS; the logistic model additionally retained associations for preoperative TACE/RFA and tumor diameter. The seven-predictor nomogram showed moderate discrimination after optimism correction (AUC 0.744), emphasizing that the model is exploratory rather than ready for clinical deployment.

The 43.3% early-recurrence proportion is high but clinically plausible for this cohort. A Western series restricted to HCC ≤2 cm reported 29% recurrence by 2 years (10), whereas an earlier ≤3-cm series reported 40% (11). Our broader ≤5-cm threshold permits greater tumor burden, and the cohort also had high prevalences of cirrhosis (83.3%), MVI (29.0%), and satellite nodules (15.0%). These factors likely contributed to the observed rate. The ≤5-cm criterion should therefore be understood as a study-specific upper-diameter definition aligned with classic surgical/transplant criteria, whereas ≤2 cm remains the conventional very-early HCC subgroup (3, 7, 8, 10).

The present model should also be positioned relative to existing work. Wang et al. developed a nomogram in 326 patients with HCC ≤3 cm using AFP, MVI M2, Edmondson–Steiner III–IV grade, non-anatomic resection, and a margin <1 cm, with reported 2-year RFS AUCs of 0.856 and 0.874 in training and split-sample validation sets (9). Our model is not proposed as superior. Its narrower contribution is the combined evaluation of continuous Ki-67, microscopic dissemination, PT, and preoperative treatment history in HCC ≤5 cm, with bootstrap correction showing that its apparent discrimination was optimistic.

Ki-67 showed the most consistent association with recurrence and reflects proliferative activity (16, 21). MVI and satellite nodules indicate microscopic dissemination and remained associated with shorter RFS after adjustment (12–15). Together, these findings explain why tumors limited in diameter can recur rapidly after apparently complete resection. PT also remained associated in both adjusted models. This may reflect impaired hepatic synthetic function or portal-hypertension-related vulnerability rather than tumor biology alone; it should be viewed as a prognostic marker, not a mechanistic cause. Preoperative ascites was retained to represent portal-hypertension status but was not independently significant in the complete-case models.

The preoperative TACE/RFA finding requires particular caution. Logistic regression addresses whether recurrence occurred by 24 months, the log-rank comparison is unadjusted, and Cox regression uses the timing and censoring of events while adjusting for other predictors. Thus, the strong adjusted logistic association (OR 6.673) can coexist with an attenuated Cox estimate (HR 1.829, *P* = 0.071). Only 17 patients received preoperative locoregional therapy, producing wide confidence intervals and unstable precision. The association is likely influenced by confounding by indication and selection of more complex or biologically aggressive tumors; no causal adverse effect of TACE/RFA can be inferred (22). In the restricted cohort, the MVI effect remained similar (HR 1.763 in the main model vs 1.688) and significant, while the tumor-size estimate increased (HR 1.171 to 1.299) and became significant. These shifts are compatible with removal of a small selected subgroup and redistribution of shared prognostic information among correlated tumor variables, rather than reversal of biological effects. More broadly, postoperative recurrence after HCC resection has been characterized with respect to risk factors, early versus late recurrence patterns, post-recurrence management, and prediction modeling (23–28).

Internal validation materially tempered the performance claim. Adding PT and ascites increased the apparent AUC from the original five-predictor value of 0.760 to 0.775, but the 1,000-resample correction reduced it to 0.744 and yielded a calibration slope of 0.808, consistent with modest overfitting. The nomogram should therefore be considered a hypothesis-generating risk stratification tool. Independent validation, recalibration, and assessment of clinical consequences are required before use in surveillance or treatment decisions (19, 20).

Missingness was not completely random. Ki-67 was missing more often in earlier years and among younger patients, making temporal ascertainment and selection bias plausible. Multiple imputation preserved the main effect directions but shifted some borderline significance results, including preoperative ascites in the Cox model. The complete-case and imputed analyses should therefore be interpreted together. Other limitations include the retrospective single-center design, potential residual confounding, routine-practice recurrence ascertainment without consistent pathological confirmation, retrospective harmonization of nonstandard differentiation descriptions, only 17 preoperative TACE/RFA exposures, and absence of external validation.

Despite these limitations, the study shows that limited diameter alone does not capture early-recurrence risk after HCC resection. Routine pathology and laboratory information—particularly Ki-67, MVI, satellite nodules, and PT—may help identify hypotheses and candidate variables for future prospectively specified, multicenter prediction studies. .

## Conclusions

Among patients undergoing hepatic resection for HCC ≤5 cm, Ki-67, MVI, satellite nodules, and PT were associated with shorter 2-year RFS. A seven-predictor exploratory nomogram showed moderate optimism-corrected discrimination. External validation and recalibration are required before clinical use.

## Data Availability

The raw data supporting the conclusions of this article will be made available by the authors, without undue reservation.
