# Supplementary material for: Clinicopathological predictors and an internally validated exploratory nomogram for 2-year recurrence after hepatic resection of hepatocellular carcinoma ≤5 cm
Source: Front Med (Lausanne). 2026 Oct 8;13:1948479. doi: 10.3389/fmed.2026.1948479 (PMC13686892; doi:10.3389/fmed.2026.1948479)
Supplement: Supplementary file 1 [file Supplementaryfile1.docx]

**Supplementary Material**

Supplementary Table 1. Sensitivity analysis after excluding patients who received preoperative TACE/RFA

| **Variable** | **N** | **Events** | **Univariable HR (95% CI)** | **Univariable P value** | **Adjusted HR (95% CI)** | **Adjusted P value** |
| --- | --- | --- | --- | --- | --- | --- |
| Ki-67 (per 1%) | 175 | 71 | 1.025 (1.014–1.036) | <0.001 | 1.027 (1.016–1.039) | <0.001 |
| Microvascular invasion | 197 | 80 | 2.014 (1.286–3.153) | 0.002 | 1.688 (1.035–2.751) | 0.036 |
| Satellite nodules | 197 | 80 | 2.598 (1.549–4.357) | <0.001 | 2.122 (1.193–3.776) | 0.010 |
| Tumor size (per cm) | 198 | 80 | 1.186 (0.988–1.423) | 0.068 | 1.299 (1.043–1.616) | 0.019 |
| Prothrombin time (per s) | 198 | 80 | 1.308 (1.097–1.561) | 0.003 | 1.233 (1.017–1.495) | 0.033 |
| Preoperative ascites | 198 | 80 | 1.597 (0.984–2.593) | 0.058 | 1.277 (0.732–2.228) | 0.390 |

Patients who received preoperative TACE/RFA were excluded. RFS was censored at 24 months. The adjusted model included Ki-67, MVI, satellite nodules, tumor diameter, PT, and preoperative ascites. HRs >1 indicate shorter 2-year RFS/higher recurrence hazard. Abbreviations: CI, confidence interval; HCC, hepatocellular carcinoma; HR, hazard ratio; MVI, microvascular invasion; RFA, radiofrequency ablation; RFS, recurrence-free survival; TACE, transarterial chemoembolization.

Supplementary Table 2. Missing-data profile for variables analyzed in Tables 1 and 2

| **Variable** | **Available, n** | **Missing, n** | **Missing, %** |
| --- | --- | --- | --- |
| Age | 215 | 0 | 0.0 |
| Sex | 215 | 0 | 0.0 |
| Cirrhosis | 215 | 0 | 0.0 |
| History of hepatitis | 215 | 0 | 0.0 |
| Antiviral treatment | 215 | 0 | 0.0 |
| Hypertension | 215 | 0 | 0.0 |
| Diabetes mellitus | 215 | 0 | 0.0 |
| Smoking | 215 | 0 | 0.0 |
| Alcohol use | 215 | 0 | 0.0 |
| Preoperative TACE/RFA | 215 | 0 | 0.0 |
| Preoperative ascites | 215 | 0 | 0.0 |
| WBC | 215 | 0 | 0.0 |
| Neutrophils | 215 | 0 | 0.0 |
| Lymphocytes | 215 | 0 | 0.0 |
| Platelets | 215 | 0 | 0.0 |
| AFP | 214 | 1 | 0.5 |
| Albumin | 215 | 0 | 0.0 |
| AST | 215 | 0 | 0.0 |
| ALT | 215 | 0 | 0.0 |
| Total bilirubin | 215 | 0 | 0.0 |
| PT | 215 | 0 | 0.0 |
| D-dimer, numeric | 185 | 30 | 14.0 |
| NLR | 215 | 0 | 0.0 |
| PLR | 215 | 0 | 0.0 |
| SII | 215 | 0 | 0.0 |
| Transfusion | 215 | 0 | 0.0 |
| Postoperative ascites | 215 | 0 | 0.0 |
| Intraoperative blood loss | 215 | 0 | 0.0 |
| Operation time | 214 | 1 | 0.5 |
| First porta hepatis occlusion time | 54 | 161 | 74.9 |
| Tumor maximum diameter | 215 | 0 | 0.0 |
| Tumor number | 215 | 0 | 0.0 |
| Ki-67 | 190 | 25 | 11.6 |
| Differentiation usable in 3-tier analysis | 202 | 13 | 6.0 |
| Capsule | 214 | 1 | 0.5 |
| MVI | 214 | 1 | 0.5 |
| Satellite nodules | 214 | 1 | 0.5 |
| Vascular invasion | 214 | 1 | 0.5 |
| Nerve invasion | 212 | 3 | 1.4 |
| CD34 IHC | 191 | 24 | 11.2 |
| GPC-3 IHC | 185 | 30 | 14.0 |
| AFP IHC | 185 | 30 | 14.0 |

For D-dimer, 29 blank entries and one nonnumeric entry were treated as missing. For the three-tier differentiation analysis, 10 reports lacked an explicit grade and three were missing. IHC, immunohistochemistry; MVI, microvascular invasion.

Supplementary Table 3. Characteristics of patients with observed versus missing Ki-67

| **Variable** | **Ki-67 observed (n=190)** | **Ki-67 missing (n=25)** | **P value** |
| --- | --- | --- | --- |
| Operation year | 2019.00 [2017.00, 2021.00] | 2017.00 [2015.00, 2019.00] | 0.012 |
| Age, years | 55.00 [49.00, 64.00] | 52.00 [42.00, 58.00] | 0.042 |
| PT, s | 12.20 [11.60, 12.90] | 12.60 [11.70, 13.90] | 0.251 |
| Tumor diameter, cm | 3.00 [2.00, 4.00] | 3.00 [2.00, 4.50] | 0.785 |
| Recurrence within 2 years | 83/190 (43.7%) | 10/25 (40.0%) | 0.831 |
| Male sex | 148/190 (77.9%) | 17/25 (68.0%) | 0.314 |
| Cirrhosis | 158/190 (83.2%) | 21/25 (84.0%) | 1.000 |
| Preoperative TACE/RFA | 15/190 (7.9%) | 2/25 (8.0%) | 1.000 |
| Preoperative ascites | 42/190 (22.1%) | 5/25 (20.0%) | 1.000 |
| MVI | 56/190 (29.5%) | 6/24 (25.0%) | 0.812 |
| Satellite nodules | 30/190 (15.8%) | 2/24 (8.3%) | 0.543 |

Continuous variables are median [IQR] and were compared by Mann–Whitney U test; binary variables are n/N (%) and were compared by Fisher's exact test. Missing Ki-67 decreased over calendar time (OR per year 0.798, 95% CI 0.670–0.951; P=0.012).

Supplementary Table 4. Multiple-imputation sensitivity analysis: logistic model for recurrence within 24 months

| **Variable** | **Complete-case OR (95% CI)** | **P value** | **MI-pooled OR (95% CI)** | **P value** |
| --- | --- | --- | --- | --- |
| Ki-67 (per 1%) | 1.028 (1.011–1.044) | <0.001 | 1.027 (1.011–1.043) | 0.001 |
| Preoperative TACE/RFA | 6.673 (1.529–29.117) | 0.012 | 4.888 (1.314–18.182) | 0.018 |
| Microvascular invasion | 2.301 (1.098–4.824) | 0.027 | 2.307 (1.132–4.701) | 0.021 |
| Satellite nodules | 2.871 (1.099–7.497) | 0.031 | 3.205 (1.258–8.165) | 0.015 |
| Tumor size (per cm) | 1.374 (1.030–1.834) | 0.031 | 1.286 (0.983–1.683) | 0.067 |
| Prothrombin time (per s) | 1.387 (1.033–1.861) | 0.030 | 1.440 (1.090–1.903) | 0.010 |
| Preoperative ascites | 1.566 (0.713–3.439) | 0.264 | 2.010 (0.952–4.244) | 0.067 |

Supplementary Table 5. Multiple-imputation sensitivity analysis: Cox model for 2-year recurrence-free survival

| **Variable** | **Complete-case HR (95% CI)** | **P value** | **MI-pooled HR (95% CI)** | **P value** |
| --- | --- | --- | --- | --- |
| Ki-67 (per 1%) | 1.022 (1.012–1.033) | <0.001 | 1.022 (1.012–1.033) | <0.001 |
| Preoperative TACE/RFA | 1.829 (0.950–3.519) | 0.071 | 1.849 (0.984–3.477) | 0.056 |
| Microvascular invasion | 1.763 (1.111–2.796) | 0.016 | 1.794 (1.150–2.797) | 0.010 |
| Satellite nodules | 1.924 (1.129–3.279) | 0.016 | 2.123 (1.275–3.536) | 0.004 |
| Tumor size (per cm) | 1.171 (0.963–1.425) | 0.114 | 1.125 (0.936–1.352) | 0.208 |
| Prothrombin time (per s) | 1.235 (1.030–1.481) | 0.023 | 1.252 (1.056–1.486) | 0.010 |
| Preoperative ascites | 1.420 (0.842–2.394) | 0.188 | 1.636 (1.005–2.662) | 0.048 |

MI, multiple imputation. Fifty imputed datasets were generated and pooled using Rubin's rules. Effect estimates >1 indicate higher odds of recurrence or shorter recurrence-free survival.
